# Free vibration of functionally graded carbon-nanotube-reinforced composite plates with cutout

**DOI:** 10.3762/bjnano.7.45

**Published:** 2016-04-07

**Authors:** Mostafa Mirzaei, Yaser Kiani

**Affiliations:** 1 Department of Mechanical Engineering, Faculty of Engineering, University of Qom, Qom, Iran; 2 Faculty of Engineering, Shahrekord University, Shahrekord, Iran

**Keywords:** Chebyshev polynomials, cutout, functionally graded carbon-nanotube-reinforced composite, Ritz method

## Abstract

During the past five years, it has been shown that carbon nanotubes act as an exceptional reinforcement for composites. For this reason, a large number of investigations have been devoted to analysis of fundamental, structural behavior of solid structures made of carbon-nanotube-reinforced composites (CNTRC). The present research, as an extension of the available works on the vibration analysis of CNTRC structures, examines the free vibration characteristics of plates containing a cutout that are reinforced with uniform or nonuniform distribution of carbon nanotubes. The first-order shear deformation plate theory is used to estimate the kinematics of the plate. The solution method is based on the Ritz method with Chebyshev basis polynomials. Such a solution method is suitable for arbitrary in-plane and out-of-plane boundary conditions of the plate. It is shown that through a functionally graded distribution of carbon nanotubes across the thickness of the plate, the fundamental frequency of a rectangular plate with or without a cutout may be enhanced. Furthermore, the frequencies are highly dependent on the volume fraction of carbon nanotubes and may be increased upon using more carbon nanotubes as reinforcement.

## Introduction

Plates with cutouts are extensively used in automotive and aircraft structures. Cutouts may be of rectangular, circular, elliptical, super elliptical or polygonal shape. Due to the complicated configuration of a plate with a cutout, there is significantly less research on plates with cutouts in comparison to those without cut-out. Depending on the application, homogeneous isotropic, composite or functionally graded plates may be perforated to fulfill a desired application.

Representing a type of novel material with fascinating electro-thermo-mechanical properties, carbon nanotubes (CNTs) have attracted increasing attention in the past decades. CNTs are a promising candidate for the reinforcement of the matrix phase in a composite. Kwon et al. [1] reported that using a powder metallurgy fabrication process, carbon-nanotube-reinforced composites (CNTRCs) may be achieved with a nonuniform distribution of CNTs through the media. This type of reinforced composite media is known as functionally graded carbon-nanotube-reinforced composite (FG-CNTRC). An overview on the properties, modeling and characteristics of FG-CNTRC beams, plates and shells is provided by Liew et al. [2]

It has been shown that the bending moment may be significantly alleviated through a functionally graded distribution of CNTs in a polymeric matrix [3]. In the five years following the discovery of this interesting feature, various investigations were reported on the mechanics of FG-CNTRC structures.

Zhu et al. [4] investigated the free vibration and static response of FG-CNTRC plates using finite element method [4]. Zhang et al. investigated the free vibration characteristics of FG-CNTRC skew plates [5], triangular plates [6] and cylindrical panels [7] using element free methods. In these works it is shown that the natural frequencies of plates and panels are affected by the distribution and volume fraction of CNTs. Zhang et al. [8] investigated the free vibration characteristics of FG-CNTRC plates resting on an elastic foundation. Lei et al. [9] investigated the free vibration of composite, laminated FG-CNTRC plates with general boundary conditions. Malekzadeh and Zarei [10] examined the free vibration characteristics of laminated plates containing FG-CNTRC layers in an arbitrary straight-sided quadrilateral shape. Malekzadeh and Heydarpour [11] investigated the free vibration and static response of laminated plates with FG-CNTRC layers using a mixed Navier-layerwise differential quadrature method. In this research, plates with all edges simply supported are considered. Natarajan et al. [12] applied a higher order shear and normal deformable plate formulation to study the static and free vibrations of single layer FG-CNTRC plates and also sandwich plates with FG-CNTRC face sheets. Wang and Shen investigated the linear and nonlinear free vibrations of a single layer FG-CNTRC plate [13] and also sandwich plates with stiff core and FG-CNTRC face sheets [14]. In this analysis, the interaction of the plate with a two parameter elastic foundation is also taken into account. Wang and Shen [15] investigated the dynamic response of FG-CNTRC plates according to the von Kármán formulation. In this research, the interaction of a two parameter elastic foundation and a thermal environment are also included. The solution method of this research is based on a two-step perturbation technique and is suitable for plates with all edges simply supported. Using a mesh-free formulation proper for arbitrary edge supports, Lei et al. [16] investigated the elasto-dynamic response of FG-CNTRC plates subjected to sudden lateral pressure. For more investigations on vibration, buckling, postbuckling, stress analysis, and nonlinear bending of FG-CNTRC plates, one may refer to [17–25].

The present research aims to investigate the free vibration characteristics of an FG-CNTRC rectangular plate containing a central, rectangular cutout. The distribution of CNTs across the plate thickness are assumed to be either uniform or nonuniform. A modified rule of mixtures approach is used to obtain the properties of the composite media. Chebyshev polynomials are used as the basic shape functions of the Ritz formulation to construct an eigenvalue problem. The solution method may be used for perforated FG-CNTRC rectangular plates with arbitrary boundary conditions on the outer edges, while the inner edges are unconstrained. The numerical results allow for the study of the volume fraction and distribution pattern of CNTs, plate boundary conditions and hole size.

## Modeling

### Basic formulation

**Figure 1:**
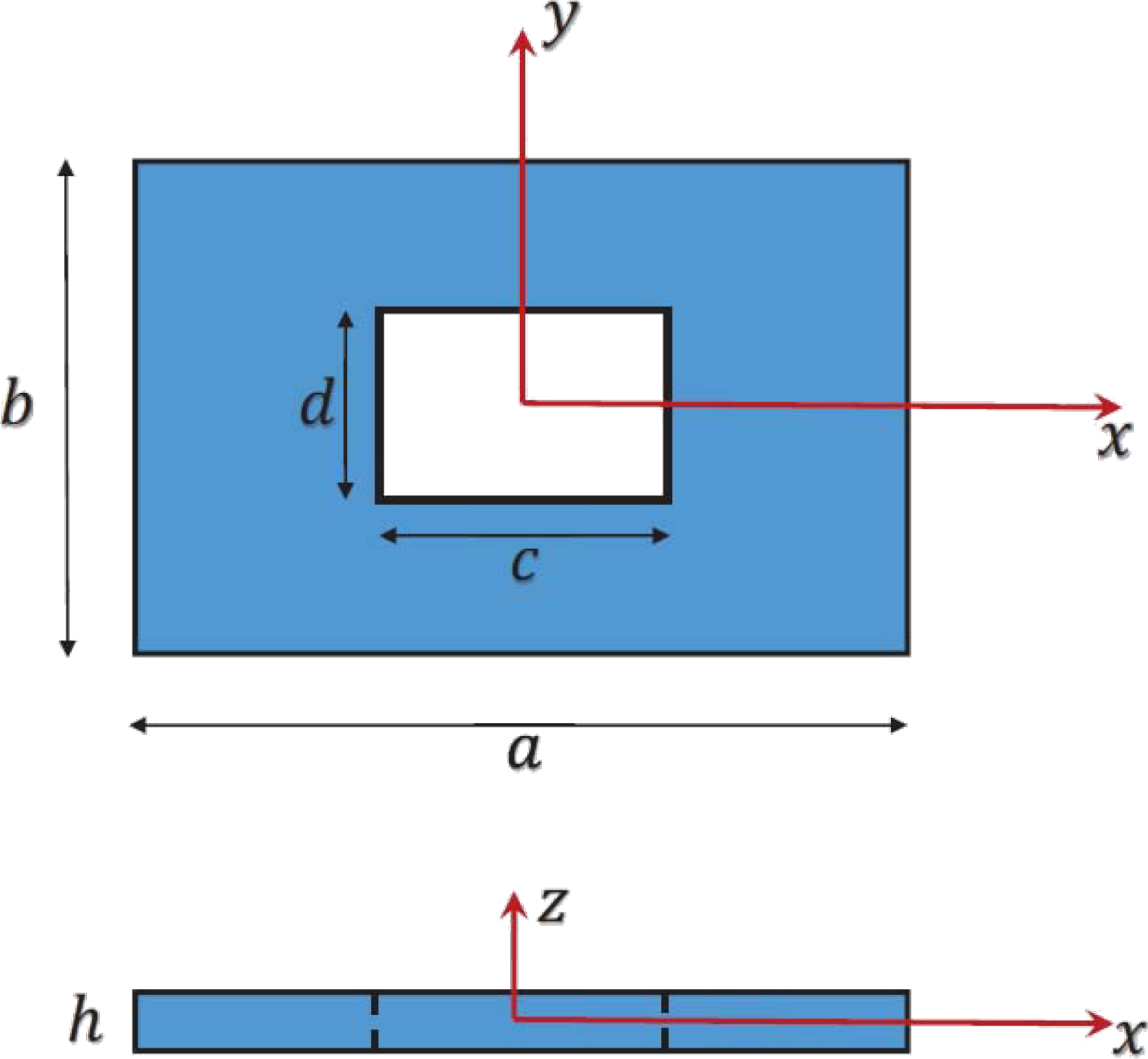
A schematic of the geometric features of the plate along with the assigned coordinate system.

A rectangular-shaped plate, made of a polymeric matrix, reinforced by CNTs whose distribution may be nonuniform, is considered in the present research. The plate contains a centered hole, which is assumed to be rectangular-shaped. The cartesian coordinate system is assigned to the center of the mid-surface of the plate. In this system, the plate occupies the domain [−0.5*a* 0.5*a*] × [−0.5*b* 0.5*b*] × [−0.5*h* 0.5*h*]. The hole occupies the domain [−0.5*c* 0.5*c*] × [−0.5*d* 0.5*d*] × [−0.5*h* 0.5*h*]. The dimensions of the plate with the assigned coordinate system are demonstrated in Figure 1.

**Figure 2:**
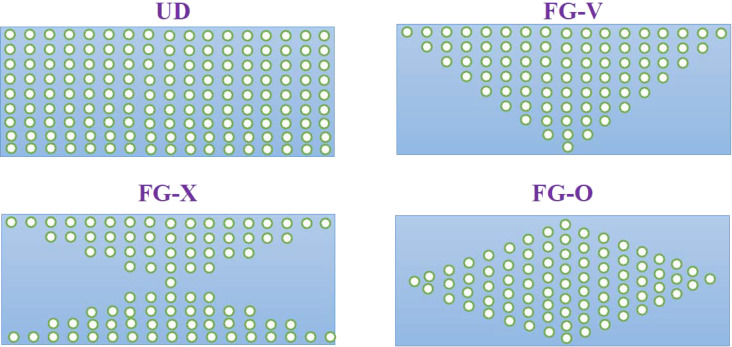
Various graded patterns of FG-CNTRC plates.

Motivated by the fundamental research of Shen [3], many investigators take into account the functionally graded distribution of the volume fraction of reinforcements through the matrix. Consistent with the possible fabrication processes for plates, three different functionally graded types of CNT dispersion profiles may be assumed and are considered in the present research: FG-V, FG-O and FG-X [5–7]. A schematic of these functionally graded types along with the uniformly distributed (UD) type are shown in Figure 2.

The properties of a composite media (i.e., a matrix reinforced with CNTs) may be obtained according to various homogenization techniques. The two commonly used schemes that are extensively used for composites and FGMs are the Mori–Tanaka scheme [26] and the rule of mixtures [27]. The conventional rule of mixtures has the advantage of simplicity; however, when using CNTRCs, this approach does not provide an accurate estimation of the mechanical properties of the media. Meanwhile, as explained by Shen [3] and used extensively by other researchers [28–32], the conventional rule of mixtures approach may be modified with the introduction of the efficiency parameters. Under such modification, Young’s modulus and the shear modulus of the composite media take the form:

[1]
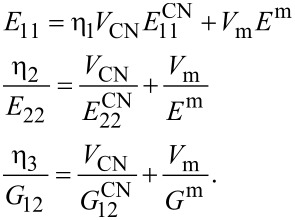


In this formula, the properties of the CNT are denoted by a superscript CN and that those belong to matrix are denoted by a superscript m. Following the classical solid mechanics notation, *E* and *G* are the elastic modulus and shear modulus of the constituents, respectively. In comparison to the conventional rule of mixtures approach, three unknown constants, η_1_, η_2_ and η_3_, are introduced in Equation 1; these are known as efficiency parameters. These parameters compensate for the errors generated due to the conventional rule of mixtures approach for a CNTRC. The values of these constants are obtained by matching the data obtained according to the above formula with those obtained based on the molecular dynamics simulation.

It is worth noting that the volume fraction of CNTs and polymeric matrix are denoted by *V*_CN_ and *V*_m_, respectively. According to the partition of unity property, the following condition should be satisfied at each point of the composite media: *V*_CN_ + *V*_m_ = 1.

The volume fraction of CNTs is assumed to be either nonuniform or uniform across the plate thickness. According to the above rule, the volume fraction of matrix may also be achieved and the overall properties of the media may be calculated according to Equation 1. Table 1 presents the dispersion profile of *V*_CN_ as a function of the thickness coordinate for each of the UD CNTRC or FG-CNTRC rectangular plates.

**Table 1:** Volume fraction of CNTs as a function of the thickness coordinate for various CNT distributions [28–34].

CNT Distribution	*V*_CN_

UD CNTRC	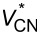
FG-V CNTRC	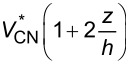
FG-O CNTRC	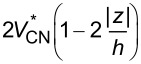
FG-X CNTRC	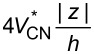

Upon evaluation of the total volume fraction of CNTs across the plate thickness, it is revealed that all types have the same total volume fraction of CNTs, that is, 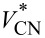. Consequently, the vibrational characteristics of FG-CNTRC and UD-CNTRC rectangular plates may be compared with respect to each other. As previously shown in Figure 2 and the information in Table 1, given an FG-X pattern of CNT dispersion, the mid-surface of the plate is free of CNTs while the top and bottom surfaces have the maximum volume fraction of CNTs. The volume fraction of CNTs increases linearly from the mid-plane to the free surfaces of the plate. The FG-O type of distribution pattern is the inverse of the FG-X case. In the FG-O distribution, the top and bottom surfaces are free of CNTs and the mid-surface has the maximum volume fraction of CNTs. In FG-V type, the bottom surface is free of CNTs and the top has the maximum volume fraction of CNTs. Unlike these three types, in the UD case, each surface of the plate has the same volume fraction of CNTs.

Similar to the shear modulus and Young’s modulus, Poisson’s ratio and the mass density of the composite media may be written in terms of belongings to the CNT and matrix. As claimed by Shen [28], and as used also by other researchers [29], Poisson’s ratio depends weakly on position and consequently may be obtained as

[2]
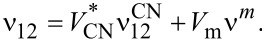


The mass density of a CNTRC media may be obtained according to the conventional rule of mixtures approach [13–14]. Therefore, as a function of volume fraction and mass density of constituents, ρ^CN^ and ρ^m^, one may write

[3]
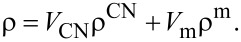


Upon evaluation of the mass fraction for each of the graded patterns of CNTs, it is concluded that each type has the same mass fraction of CNTs.

Flexural theories propose an approximate function for the in-plane and out-of-plane displacement components of the plate. The most simple flexural theory is the classical plate theory, which eliminates the transverse shear strain components as well as the normal strain component. These assumptions are exaggerated for moderately thick composites and therefore classical plate theory results in erroneous results for the structural response of a CNTRC rectangular plate. On the other hand, first order shear deformation plate theory (FSDT), which takes into account the constant transverse shear strain, results in accurate results for the global properties of moderately thick CNTRC plates. This is because it takes into account both the rotary inertias and through-the-thickness shear strains [35]. This research is also developed based on FSDT, which estimates the displacements of the plate in terms of those of the mid-surface and the cross-section rotations as

[4]
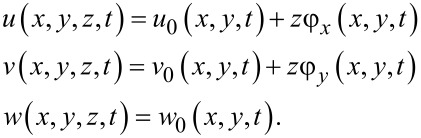


In Equation 4, the subscript zero indicates the characteristics of the mid-plane. Rotations of the cross-sectional elements about the *x* and *y* axes are denoted by φ*_y_* and φ*_x_*. Additionally, displacements along the *x*, *y* and *z* directions are shown by *u*, *v* and *w*.

The substitution of Equation 4 into the strain–displacement relations results in the components of strain on an arbitrary point of the plate in terms of mid-surface strain components and change in curvature as

[5]
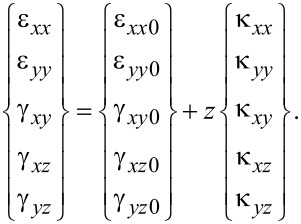


The strain field on the midsurface of the plate may be obtained according to the midsurface displacements as

[6]
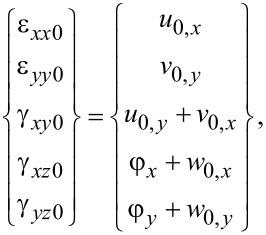


and the change of curvatures may be obtained in terms of cross-section rotations as

[7]
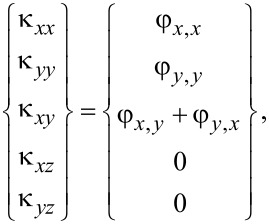


where in Equation 6 and Equation 7 (and hereafter), the comma in the subscript indicates the derivative with respect to the variable following the comma.

Under linear elastic behavior of the composite media, the strain components may be obtained in terms of strain components according to the following generalized Hook law as

[8]
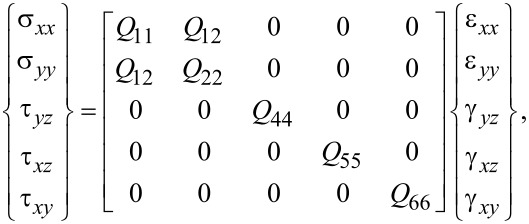


where the plane-stress stiffnesses of the plate are denote by *Q_ij_* components (*i,j* = 1,2,4,5,6). These constants may be obtained in terms of the Poisson’s ratio, shear modulus and Young’s modulus of the composite plate as [29]

[9]
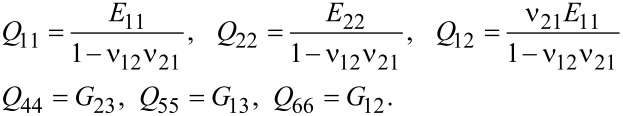


To construct the motion equations of the plate, the Hamilton principle may be used [35]. For free vibration analysis where external forces/moments are absent, Hamilton’s principle may be written as

[10]
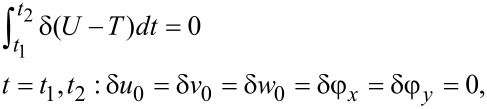


where δ*U* is the virtual strain energy of the perforated plate which may be calculated as

[11]
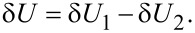


In the above equation and in the rest of this work, the subscripts 1 and 2 denote a solid rectangle (i.e., a solid rectangle without a cutout) and the cutout segment, respectively. The strain energies may be obtained upon integration of the density of the strain energy over the suitable volume.

[12]
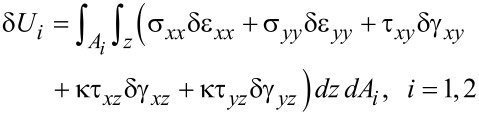


where the shear correction factor is denoted by κ. This parameter is used to compensate for the errors due to the assumption of constant shear strains across the thickness. The exact value of this factor is not straightforward and may be obtained under evaluation of complicated integrals. Since the exact value of this factor depends on the boundary conditions, geometry of the media, material and loading, the approximate value of κ = 5/6 is used in the present research.

Similarly, δ*T* is the variation of the kinetic energy of the plate which also may be written as

[13]
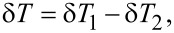


where the kinetic energy may be obtained as

[14]
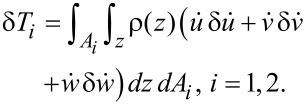


### Solution procedure

It is known that the equations of motion for a plate with three translational motion and two rotational motion components may be achieved using the process of virtual displacements with the aid of the Green–Gauss theorem. On the other hand, the matrix representation of the equations of motion may be established using the application of energy methods to Equation 10. As one of the most widely known energy-based methods, the Ritz method is used in the present research. The effectiveness and efficiency of various types of Ritz methods has been the subject of many studies [36–39]. In this study, the approximation of the displacement field is carried out using the Ritz method whose shape functions are written in terms of the Chebyshev polynomials. As a result, the essential variables may be written as

[15]
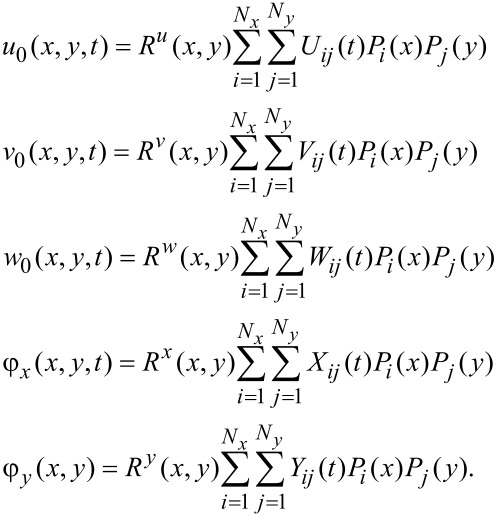


In Equation 15, the *i*-th Chebyshev polynomial of the first kind is denoted by *P_i_*. These functions in a closed-form expression may be written as

[16]
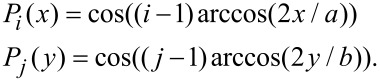


Additionally, in Equation 15, the auxiliary functions (*R*^α^(*x,y*), where α = *u,v,w,x,y*) are called the boundary functions, which are associated with the essential boundary conditions. It is known that in the Ritz method, the shape functions should at least satisfy the essential boundary conditions.

Three types of mechanical boundary conditions are widely used for each of the edges of the plate: clamped (C), simply supported (S) and free (F) edges are the assumed types of boundary conditions in the present study. For a clamped edge, three components of the displacement field and two components of the rotation should be zero at the edge. For a simply supported one, the tangential displacement, tangential rotation and lateral displacement should be zero. Finally, for a free edge, none of the boundary conditions are applied, and therefore, none of the displacements and rotations are restrained at the edge. On each exterior edge of the plate, various boundary conditions may be defined; however, the interior edges are all assumed to be free and none of the boundary conditions around the hole are applied.

Since the Chebyshev polynomials of the fist kind are nonzero on both ends of the interval (i.e., *P_i_*(±1) ≠ 0), the auxiliary functions *R*^α^, α = *u,v,w,x,y* should be chosen to satisfy the essential boundary conditions on the edge when necessary. Each of the auxiliary functions *R*^α^, α = *u,v,w,x,y* may be written generally as

[17]
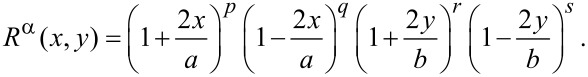


The newly introduced parameters, *p*, *q*, *r* and *s*, are equal to zero or one and their magnitude depends on the essential boundary conditions at the edge. As an example, consider a perforated plate with clamped boundary conditions at *x* = −0.5*a* and *x* = +0.5*a*, free at *y* = −0.5*b*, and simply supported at *y* = +0.5*b*. For such a case, the auxiliary functions (*R*^α^, where α = *u,v,w,x,y*) are given as

[18]
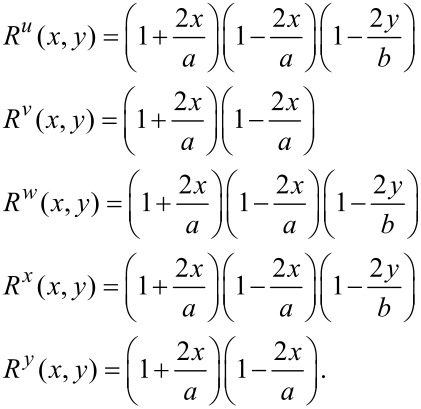


Finally, the substitution of the series expansion of Equation 15 into Equation 12 and Equation 14, and inserting the results into the Hamilton principle of Equation 10 results in the motion equations given as

[19]
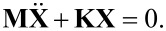


In the above equation, **M** is the mass matrix and, **K** is the stiffness matrix. Additionally, the mechanical displacement vector is denoted by **X**, which consists of the unknown displacements *U_ij_*, *V_ij_*, *W_ij_*, *X_ij_* and *Y_ij_*.

Since the free vibration response is under investigation, **X** = 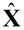 sin(*ω t+φ*) may be considered, where ω is the natural frequency. The substitution of this equation into Equation 19 results in an eigenvalue problem as

[20]
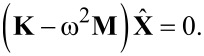


This eigenvalue problem can be solved using the standard eigenvalue algorithms provided in a Matlab code. It is worth noting that trapezoidal numerical integration is used to evaluate the elements of the mass and stiffness matrices. In numerical integration, the interval is divided into 100 segments.

## Results and Discussion

The free vibration characteristics of FG-CNTRC rectangular plates with a centric rectangular hole were formulated in the previous sections. In the following, to assure the effectiveness and accuracy of the presented solution method, convergence and comparison studies are carried out. Next, parametric studies are provided to explore the effects of carbon nanotube characteristics on the frequencies of the perforated plate. The following convention is established for boundary conditions herein and is used in the rest of this work. For instance, an SCFS plate indicates a plate which is simply supported at *x* = −0.5*a* and *y* = +0.5*b*, clamped at *y* = −0.5*b*, and free at *x* = +0.5*b*.

In the numerical results of the present research, isotropic poly(methyl methacrylate), referred to as PMMA, is selected as the polymeric matrix. The mechanical properties of the PMMA are *E*^m^ = 2.5 GPa, ν^m^ = 0.34 and ρ^m^ = 1150 kg/m^3^. Reinforcement of the matrix is chosen as (10,10)-armchair SWCNT. For this kind of reinforcement, which is orthotropic, the material properties are given as 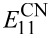 = 5.6466 TPa, 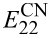 = 7.0800 TPa, *G*_12_ = 1.9445 TPa, ν = 0.175 and ρ = 1400 kg/m^3^ [40].

Finally, the efficiency parameters should be known to obtain the overall properties of the composite media, which are the stretching, coupling and bending stiffnesses. As mentioned before, these parameters are obtained by matching the data obtained by the present modified rule of mixtures approach and the molecular dynamics simulations of other researchers. A molecular dynamics simulation was performed by Han and Elliott [41]; however, since the condition of maximum thickness for CNTs was not satisfied in this research, their simulations were re-examined by Shen [28]. In the simulations of Han and Elliott [41], the effective thickness of the CNTs is set equal to at least 0.34 nm, which is open to criticism since it violates the criteria proposed by Wang and Zhang [42]. The molecular dynamics simulations of Shen [28] result in the following efficiency parameters for the CNTRC media that depend on the volume fraction of CNTs: η_1_ = 0.137 and η_2_ = 1.022 for 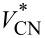 = 0.12; η_1_ = 0.142 and η_2_ = 1.626 for 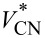 = 0.17; and η_1_ = 0.141 and η_2_ = 1.585 for 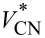 = 0.28. For each case, the efficiency parameter η_3_ is equal to 0.7η_2_. The shear modulus *G*_13_ is taken equal to *G*_12_, whereas *G*_23_ is taken equal to 1.2*G*_12_ [28].

### Convergence and comparison studies

Convergence and comparison studies are presented in this section. First, the convergence study allows for the necessary shape functions to be obtained with the series expansion of the Ritz method, with results shown in Table 2. In this study, the first three frequency parameters of a square plate with a square cutout at the center are evaluated in terms of the number of shape functions. Two different cutout sizes are considered. The results are also compared with those of Liew et al. [43] and Lam et al. [44]. In the solution method of Liew et al. [43], the basic L-shaped element, which is divided into appropriate sub-domains that are dependent upon the location of the cutout, is used as the basic building element. Lam et al. [44], on the other hand, obtained the frequencies according to a Ritz method whose shape functions are generated using the Gram–Schmidt process. In both of the above-mentioned references, the plate is formulated using the classical plate theory and for the sake of comparison, in the present analysis, the side-to-thickness ratio is chosen as *a/h* = 100. It is seen that the results of our study match well with those of Liew et al. [43] and Lam et al. [44] after the adoption of *N_x_* = *N_y_* = 20 shape functions. Therefore, in the subsequent results, the number of shape functions in both directions is chosen as 20.

**Table 2:** Convergence study on the first three frequency parameters 
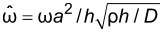
 of SSSS isotropic homogeneous square plates with *a/h* = 100, ν = 0.3 and two cutout ratios.

*N_x_* = *N_y_*	*c/a=0.5*			*c/a=0.3*		
	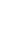	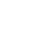	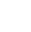	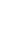	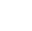	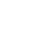

4	25.3219	67.0738	96.5711	21.2056	59.9002	91.5117
6	24.3337	52.4935	79.6489	20.7782	49.9952	76.7551
8	23.9120	48.0090	76.8117	20.3092	49.4326	76.0125
10	23.7717	44.2278	74.2547	19.9607	48.9185	75.6918
12	23.7394	42.6920	72.9483	19.8747	48.1375	75.3304
14	23.7177	42.1001	72.4587	19.8625	47.2216	74.9326
16	23.6514	41.6152	72.1370	19.7767	46.4394	74.6078
18	23.5996	41.2933	71.8660	19.7260	45.9450	74.3541
20	23.5641	41.0550	71.7298	19.6490	45.5670	74.2122
Liew et al. [43]	23.441	41.779	71.737	19.391	44.799	73.656
Lam et al. [44]	23.235	39.712	69.868	19.357	44.207	73.906

In Table 3, the first four frequencies of a plate with a centric cutout clamped all around is evaluated. In this study, the plate is also a square, and for the sake of comparison, the side-to-thickness ratio is chosen as *a/h* = 100. Four different square cutout sizes, *c/a* = 0.1, 0.2, 0.3 and 0.5, are considered and in each case our results are compared with those of Malekzadeh et al. [45] and Mundkur et al. [46]. Malekzadeh et al. [45] obtained the frequencies according to a three dimensional elasticity formulation and using the Chebyshev–Ritz formulation, whereas boundary characteristics of orthogonal polynomial functions are invoked into the Ritz formulation by Mundkur et al. [46] to obtain the plate frequencies. It is seen that our results are in good agreement with those of both Malekzadeh et al. [45] and Mundkur et al. [46].

**Table 3:** First four frequency parameters 
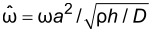
 for square CCCC isotropic homogeneous plates with ν = 0.3, *a/h* = 100 and various square cutout sizes.

*c/a*	Source	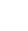	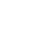	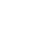	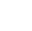

0.1	Malekzadeh et al. [45]	36.7943	73.9968	74.0389	108.1382
	Mundkur et al. [46]	36.5045	73.4142	73.4142	107.3528
	Present	36.3141	73.2476	73.2476	106.9850
0.2	Malekzadeh et al. [45]	37.9162	73.8299	73.8882	105.9458
	Mundkur et al. [46]	38.1073	73.6267	73.6267	105.4715
	Present	37.2017	72.7578	72.7578	104.7691
0.3	Malekzadeh et al. [45]	41.6279	71.2093	71.3769	103.6814
	Mundkur et al. [46]	41.7912	73.9799	73.9799	104.3388
	Present	40.9624	69.0943	69.0943	101.9502
0.5	Malekzadeh et al. [45]	66.5457	79.1407	79.2248	109.2086
	Mundkur et al. [46]	65.7150	81.6796	81.6796	110.8569
	Present	65.3050	77.7074	77.7074	107.5626

Table 4 presents the frequencies of a thin square plate that is simply supported all around and contains a square cutout at the center. The cutout size is *c/a* = 0.4 and for the sake of comparison, the side-to-thickness ratio of the square plate is chosen as *a/h* = 100. The results of this study are compared with those of Liew et al. [43]. In the tabulated results, SS indicates the double-symmetric modes and AA indicates the double-antisymmetric modes. On the other hand, modes that are symmetric in one direction and antisymmetric on the other direction are denoted by AS. Again, it is seen that the results of our study are in good agreement with the available data, which verifies the accuracy of the present method.

**Table 4:** Frequency parameters, 
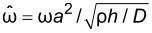
 for square SSSS isotropic homogeneous plates with a square cutout with ν = 0.3, *c/a* = 0.4 and *a/h* = 100.

Mode Type	Source	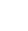	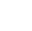	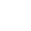

SS	Liew et al. [43]	20.7240	85.4180	136.2900
	Present	20.9151	85.8040	136.1697
AS	Liew et al. [43]	41.9070	118.7200	181.7200
	Present	42.1561	119.6766	177.3160
AA	Liew et al. [43]	71.4990	189.3300	200.9000
	Present	71.9878	188.1986	198.4664

The next comparison study gives the frequency parameters of the FG-CNTRC plate with clamped boundary conditions. The frequencies are evaluated from the proposed approach of our study and compared with those given by Zhu et al. [4] based on the finite elements method. It is worth noting that in the analysis of Zhu et al. [4], the matrix is made from PmPV with elasticity modulus *E*^m^ = 2.1 GPa, Poisson’s ratio ν^m^ = 0.34 and mass density ρ^m^ = 1150 kg/m^3^. The volume fraction of CNTs is set equal to 0.17 and the dispersion pattern of the CNTs is of the FG-V type. In such case, the efficiency parameters are obtained as η_1_ = 0.149 and η_2_ = η_3_ = 1.381 [4]. Furthermore, *G*_23_ = *G*_13_ = *G*_12_ is assumed [4]. The frequency parameter is defined as 
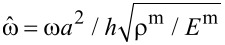
 as shown in Table 5. As can be seen, the first six frequencies are in good agreement with those obtained by Zhu et al. [4].

**Table 5:** First six natural frequencies 
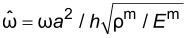
 of square CCCC FG-CNTRC plates without cutout and various side-to-thickness ratios.

	*a/h* = 10		*a/h* = 20		*a/h* = 50	

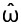	Present	Zhu et al. [4]	Present	Zhu et al. [4]	Present	Zhu et al. [4]
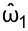	21.4953	21.544	32.5463	32.686	41.7819	42.078
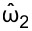	28.4093	28.613	38.9996	39.279	47.7825	48.309
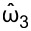	41.2024	41.431	53.4057	54.560	62.3669	63.755
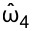	41.2818	42.119	69.5133	70.149	86.1407	90.293
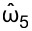	45.5711	45.796	73.3744	73.926	104.7524	106.513
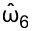	46.9814	47.055	75.1651	78.522	108.3582	110.055

The next comparison study is devoted to the case of a nonsquare plate with a nonsquare cutout. A thin plate with *a/h* = 100 and CSCS boundary conditions is considered. The length-to-width ratio is equal to *a/b* = 1.125. The cutout dimensions are the same as those of Liew et al. [43], that is, *c/a* = 1/3 and *d/b* = 1/3. The first four frequencies of the plate are obtained and compared with the available data in the literature. It is worth noting that, in this case, the experimental results of Aksu and Ali [47] are also available. A comparison is provided in Table 6. It is seen that the results of our study match well with the available data in the literature.

**Table 6:** First four frequency parameters, 
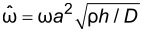
, for a CSCS, rectangular, isotropic, homogeneous plate with a rectangular cutout with ν = 0.3, *c/a* = *d/b* = *1/3*, *a/b* = *9/8* and *a/h* = 100.

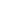	Liew et al. [43]	Aksu et al. [47] (Exp.)	Aksu et al. [47]	Lam et al. [44]	Present

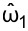	32.425	33.22	33.83	34.04	31.2802
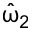	53.426	53.01	53.99	54.57	54.2069
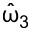	62.353	61.91	62.49	65.05	60.0453
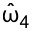	94.839	91.87	95.03	95.38	92.0645

Table 7 presents the fundamental and second symmetric modes of the frequency parameters of a unidirectional, orthotropic plate in a square platform with a centric square cutout. The material properties of the layer are *E*_11_ = 140 GPa, *E*_22_ = 3.5 GPa, *G*_12_ = 0.5 GPa, ν_12_ = 0.25 and ρ = 4000 kg/m^3^. The plate is simply supported all around and a cutout size is chosen as *c/a* = 0.5. The results are provided for various side-to-thickness ratios. A comparison is made between the results of our study with those obtained by Reddy [48] based on the finite elements method and by Ovesy and Fazilati [49] based on the finite strip method. The results are provided in Table 7. It can be seen that the results of our study match well with the available data in the literature, which proves the correctness of the formulation and solution method of the present research.

**Table 7:** Fundamental and second symmetric mode frequency parameters, 
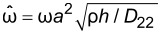
, for a SSSS, square, unidirectional, orthotropic plate with a square cutout with *c/a* = *d/b* = 1/2 and various *a/h* ratios.

	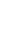			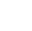		
*h/a*	Reddy [48]	Ovesy and Fazilati [49]	Present	Reddy [48]	Ovesy and Fazilati [49]	Present

0.010	51.232	51.608	51.4407	112.220	111.399	112.8712
0.040	48.907	49.049	49.0386	103.430	102.478	103.4018
0.050	47.934	47.975	47.9682	100.100	99.129	99.8877
0.100	42.693	42.108	42.0505	83.451	82.654	82.4266
0.200	34.069	32.416	32.1979	59.074	59.071	60.7709

### Parametric studies

After validating the formulation and proposed method of the present research, the parametric studies are provided in this section. In this section, the frequency parameter is defined as 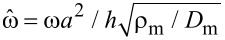, where *D*_m_ is the flexural rigidity of a plate made from the polymeric matrix.

Tables 8–11 present the first five frequencies of CNTRC plates in a square shape and side-to-thickness ratio of *a/h* = 20. Table 8, Table 9, Table 10 and Table 11 are associated with CCCC, CFFF, SSSS and CFCF plates, respectively. The volume fraction of CNTs is chosen as 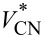 = 0.17. In each case, the frequencies are provided for three different perforation sizes and four different graded patterns of CNTs. It is seen that, similar to the case of plates without a cutout, in plates with a hole, FG-X also has the highest fundamental frequency and FG-O has the lowest. The influence of hole size on fundamental frequency is not monotonic. For instance, in CCCC plates, the fundamental frequency of a plate increases when the hole size increases from *c/a* = 0.1 to 0.3 and 0.5. This conclusion is qualitatively compatible with the results of Malekzadeh et al. [45] for CCCC FGM plates. For SSSS and CFFF plates, on the other hand, the trend is the inverse and the fundamental frequency of a plate decreases when the hole size increases from *c/a* = 0.1 to 0.3 and 0.5. The results presented in Tables 8–11 contain both the flexural and extensional as well as coupled (in FG-V type) vibrational modes. As seen from Table 10, the fourth and fifth frequencies of SSSS plates without a cutout or with a cutout size of *c/a* = 0.1 and 0.3 are the same. These frequencies are in-plane modes and, due to the symmetry of geometry and boundary conditions, they are equal. It is seen that the in-plane frequencies of FG-X and FG-O plates are equal.

**Table 8:** First five natural frequency parameters for square FG-CNTRC CCCC plates with a centric cutout. Geometrical characteristics of the plate are *a/b* = 1, *h/a* = 0.05 and various *c/a* ratios. The volume fraction of CNTs is set equal to 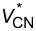 = 0.17.

*c/a*	Type	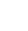	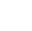	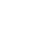	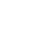	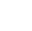

0.0	UD	104.7581	127.4624	177.3348	216.4439	229.8165
	FG-X	112.9857	136.1313	187.5684	228.1375	241.8078
	FG-O	90.1519	114.9774	166.5357	195.1187	209.9151
	FG-V	97.1637	122.2427	175.0384	205.6517	220.5951
0.1	UD	105.4667	127.4527	178.2958	210.3946	229.2000
	FG-X	114.0739	136.1672	188.9056	221.1219	241.2009
	FG-O	90.3317	114.9383	167.1189	190.7122	209.3028
	FG-V	97.5556	122.2235	175.8912	200.6315	219.9939
0.3	UD	120.4439	126.9656	169.8070	188.2571	218.9324
	FG-X	130.6054	136.3973	181.1849	199.9691	230.5113
	FG-O	102.7080	112.4737	150.9616	174.3439	199.9412
	FG-V	111.1976	120.2882	160.6058	183.8592	210.3512
0.5	UD	144.3419	145.0951	220.7844	229.0503	231.6397
	FG-X	155.3892	156.2246	233.7825	242.7781	244.6759
	FG-O	129.1618	129.9906	196.1895	205.0887	209.8574
	FG-V	137.8894	138.7569	208.3156	208.3156	217.4388

**Table 9:** First five natural frequency parameters for square, FG-CNTRC, CFFF plates with a centric cutout. Geometrical characteristics of the plate are *a/b* = 1, *h/a* = 0.05 with various *c/a* ratios. The volume fraction of CNTs is set equal to 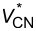 = 0.17.

*c/a*	Type	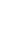	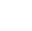	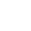	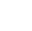	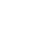

0.0	UD	22.7727	24.3214	40.0851	69.2495	83.6431
	FG-X	27.0842	28.4568	44.0066	69.7239	89.4504
	FG-O	16.7435	18.7782	36.1389	69.7239	79.6264
	FG-V	19.1381	21.0721	38.7339	69.6796	84.0389
0.1	UD	22.6504	24.2757	40.0027	68.9550	83.8138
	FG-X	26.9308	28.4058	43.8532	69.4269	89.3451
	FG-O	16.6641	18.7287	36.0570	69.4269	79.5320
	FG-V	19.0430	21.0212	38.6399	69.3808	83.9355
0.3	UD	20.4172	24.0224	37.9453	65.3384	81.1811
	FG-X	24.0635	28.1470	41.3325	65.7754	86.5017
	FG-O	15.2671	18.4600	34.5625	65.7754	76.7925
	FG-V	17.3638	20.7497	36.9148	65.6852	81.0431
0.5	UD	16.9516	23.0500	34.9990	55.9159	75.0937
	FG-X	19.6883	27.0578	38.2949	56.2537	79.9457
	FG-O	12.9576	17.6252	31.7571	56.2537	70.7251
	FG-V	14.6594	19.8497	33.9178	56.0921	74.7579

**Table 10:** First five natural frequency parameters for square, FG-CNTRC, SSSS plates with a centric cutout. Geometrical characteristics of the plate are *a/b* = 1, *h/a* = 0.05 with various *c/a* ratios. The volume fraction of CNTs is set equal to 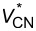 0.17.

*c/a*	Type	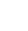	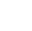	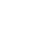	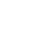	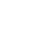

0.0	UD	63.2598	83.2741	132.0746	143.7036	143.7036
	FG-X	72.8708	92.2430	141.7590	144.7344	144.7344
	FG-O	49.4292	72.4990	123.2571	144.7344	144.7344
	FG-V	55.2524	78.2905	130.3768	144.7268	144.7268
0.1	UD	62.8020	83.1849	131.8828	144.8261	144.8261
	FG-X	72.4414	92.1454	141.5884	145.8609	145.8609
	FG-O	49.0286	72.4102	123.0916	145.8609	145.8609
	FG-V	54.8235	78.1925	130.2147	145.8510	145.8510
0.3	UD	52.8233	78.3506	111.6736	130.6302	154.9683
	FG-X	60.5716	87.1986	119.1929	139.3437	156.0783
	FG-O	41.9863	67.4468	100.7698	123.2779	156.0783
	FG-V	46.7420	73.0816	107.2829	129.9159	156.0612
0.5	UD	49.7695	72.2115	75.6430	110.4459	153.9671
	FG-X	56.2066	80.5715	80.8909	118.0424	165.6159
	FG-O	40.9160	31.2728	69.6564	103.1278	136.3056
	FG-V	45.0900	66.7584	73.9966	108.6995	146.1801

**Table 11:** First five natural frequency parameters for square, FG-CNTRC, CFCF plates with a centric cutout. Geometrical characteristics of the plate are *a/b* = 1, *h/a* = 0.05 with various *c/a* ratios. The volume fraction of CNTs is set equal to 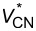 0.17.

*c/a*	Type	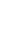	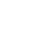	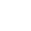	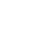	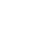

0.0	UD	100.1209	100.5478	106.0262	130.1245	142.0229
	FG-X	108.3648	108.7314	114.1163	138.8282	143.0241
	FG-O	84.7084	85.3698	92.0274	118.2125	143.0241
	FG-V	91.7731	92.3623	98.8950	125.5211	142.9107
0.1	UD	100.2534	100.3429	106.3842	129.9112	143.1875
	FG-X	108.5295	108.6060	114.7279	138.6133	144.1970
	FG-O	84.6851	85.1671	92.0179	117.9871	144.1970
	FG-V	91.8120	92.1557	99.0190	125.2861	144.0832
0.3	UD	100.7600	101.2200	118.8467	128.0103	153.1897
	FG-X	108.9985	109.4736	128.8350	137.2623	154.2720
	FG-O	85.4922	85.9562	101.4103	114.2352	151.0984
	FG-V	92.5250	93.0347	109.7041	121.8601	154.1448
0.5	UD	101.6312	101.6706	134.2600	134.9668	164.4286
	FG-X	109.8563	109.9006	144.0598	144.7920	165.5952
	FG-O	86.8830	86.5131	118.6095	119.3833	165.5945
	FG-V	93.5251	93.5551	126.8153	127.6207	165.3665

Table 12 presents the first five frequencies (including both in-plane and out-of-plane) of square plates made of FG-CNTRC with centric cutouts of various sizes. The side-to-thickness ratio is set equal to *a/h* = 20 and the plate is clamped all around. Numerical results are given for three different volume fractions of CNTs and four different graded patterns of CNTs. Similar to the case of plates without a cutout, an increase in the CNT volume fraction yields a higher natural frequency of the plate. The plates with an FG-X pattern of CNTs have higher frequencies in comparison to UD, FG-V and FG-O plates.

**Table 12:** First five natural frequency parameters for square, FG-CNTRC, CCCC plates with a centric cutout. The geometrical characteristics of the plate are *a/b* = 1, *h/a* = 0.05 and *c/a* = 0.5.

*c/a*	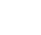	Type	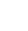	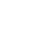	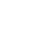	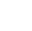	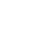

0.1	0.12	UD	83.9188	100.3679	139.1968	165.6225	180.1856
		FG-X	89.8427	105.9079	145.2992	172.7141	187.9588
		FG-O	72.7097	91.3377	131.5790	151.3920	165.3920
		FG-V	78.0171	96.2557	136.8848	158.4278	173.2206
	0.17	UD	105.4667	127.4527	178.2958	210.3946	229.2000
		FG-X	114.0739	136.1672	188.9056	221.1219	241.2009
		FG-O	90.3317	114.9383	167.1189	190.7122	209.3028
		FG-V	97.5556	122.2235	175.8912	200.6315	219.9939
	0.28	UD	117.9367	139.0787	190.6561	229.2992	249.0083
		FG-X	128.1334	152.0944	210.3997	243.4859	266.0974
		FG-O	103.9972	126.0241	176.2058	214.6312	232.7102
		FG-V	111.7471	135.8179	190.8244	224.2330	244.4012
0.3	0.12	UD	95.8721	100.3370	134.1470	147.7287	172.0447
		FG-X	102.8278	106.5212	141.7684	154.5267	179.4637
		FG-O	82.7712	89.7543	120.3341	137.5941	158.4212
		FG-V	89.0013	95.2007	127.2195	143.6480	165.5769
	0.17	UD	120.4439	126.9656	169.8070	188.2571	218.9324
		FG-X	130.6054	136.3973	181.1849	199.9691	230.5113
		FG-O	102.7080	112.4737	150.9616	174.3439	199.9412
		FG-V	111.1976	120.2882	160.6058	183.8592	210.3512
	0.28	UD	134.7884	139.7667	186.8796	202.9367	237.6707
		FG-X	147.0185	152.8559	201.8094	222.8837	254.4027
		FG-O	118.3189	125.0040	169.6503	186.3970	221.8681
		FG-V	127.5548	135.0123	180.5800	201.1201	233.5064
0.5	0.12	UD	114.3763	114.9769	174.3637	180.8571	182.4758
		FG-X	121.0992	121.7038	182.9347	189.6084	190.7261
		FG-O	102.9381	103.5831	156.5728	163.3712	166.6608
		FG-V	108.9502	109.6062	165.2573	172.0949	174.7810
	0.17	UD	144.3419	145.0951	220.7844	229.0503	231.6397
		FG-X	155.3892	156.2246	233.7825	242.7781	244.6759
		FG-O	129.1618	129.9906	196.1895	205.0887	209.8574
		FG-V	137.8894	138.7569	208.3156	208.3156	217.4388
	0.28	UD	158.9989	159.7737	242.3844	251.0307	252.5998
		FG-X	173.8958	174.8389	258.3101	268.3936	270.2030
		FG-O	143.1568	143.9204	223.0877	231.1761	234.5069
		FG-V	154.2322	155.1098	234.8549	244.0462	247.0420

## Conclusion

The natural frequencies of carbon-nanotube-reinforced, composite laminated plates with a rectangular hole in the center was analyzed in this research. The properties of the plate were obtained according to a modified rule of mixtures, which includes the efficiency parameters to account for the size-dependent characteristics of the nanocomposite. The distribution of CNTs across the plate thickness was both uniform or functionally graded. An energy-based Ritz formulation was constructed to obtain the frequencies of the plate. The basis shape functions were obtained using the Chebyshev polynomials, suitable for arbitrary in-plane and out-of-plane boundary conditions on the exterior and the cutout is assumed to be free. After performing comparison studies for isotropic and unidirectional plates with a centric cutout, the parametric studies were given for plates both with and without a cutout. It is shown that, similar to FG-CNTRC plates without a cutout, increasing the CNT volume fraction results in higher frequencies of the plate with a cutout. Furthermore, FG-X plates have a higher natural frequency in comparison to the other three patterns of CNTs. It was also demonstrated that the variation of fundamental frequency of a perforated plate with respect to the hole size is not monotonic and is dependent on the boundary conditions.
